# Designing a workplace return-to-work program for occupational low back pain: an intervention mapping approach

**DOI:** 10.1186/1471-2474-10-65

**Published:** 2009-06-09

**Authors:** Carlo Ammendolia, David Cassidy, Ivan Steensta, Sophie Soklaridis, Eleanor Boyle, Stephanie Eng, Hamer Howard, Bains Bhupinder, Pierre Côté

**Affiliations:** 1 Centre for Research Expertise in Improved Disability Outcomes (CREIDO), University Health Network, Toronto, Canada; 2 Rehabilitation Solutions, University Health Network, Toronto, Canada; 3 Department of Health Policy, Management and Evaluation, University of Toronto, Toronto, Canada; 4 Dalla Lana School of Public Health, University of Toronto, Toronto, Canada; 5 Institute for Work & Health, Toronto, Canada

## Abstract

**Background:**

Despite over 2 decades of research, the ability to prevent work-related low back pain (LBP) and disability remains elusive. Recent research suggests that interventions that are focused at the workplace and incorporate the principals of *participatory ergonomics *and return-to-work (RTW) coordination can improve RTW and reduce disability following a work-related back injury. Workplace interventions or programs to improve RTW are difficult to design and implement given the various individuals and environments involved, each with their own unique circumstances. Intervention mapping provides a framework for designing and implementing complex interventions or programs. The objective of this study is to design a best evidence RTW program for occupational LBP tailored to the Ontario setting using an intervention mapping approach.

**Methods:**

We used a qualitative synthesis based on the intervention mapping methodology. Best evidence from systematic reviews, practice guidelines and key articles on the prognosis and management of LBP and improving RTW was combined with theoretical models for managing LBP and changing behaviour. This was then systematically operationalized into a RTW program using consensus among experts and stakeholders. The RTW Program was further refined following feedback from nine focus groups with various stakeholders.

**Results:**

A detailed five step RTW program was developed. The key features of the program include; having trained personnel coordinate the RTW process, identifying and ranking barriers and solutions to RTW from the perspective of all important stakeholders, mediating practical solutions at the workplace and, empowering the injured worker in RTW decision-making.

**Conclusion:**

Intervention mapping provided a useful framework to develop a comprehensive RTW program tailored to the Ontario setting.

## Background

Back pain continues to be the leading cause of morbidity and lost productivity in the workplace [1,2]. Despite over two decades of research, the ability to prevent work-related low back pain (LBP) disability remains elusive [3]. This is particularly true in Ontario, where there has been an alarming increase in the duration of disability following occupational LBP. From 1998 to 2005 the Workplace Safety & Insurance Board (WSIB) reported a 38% increase in the proportion of injured workers who remain on benefits at 12 months, with LBP the most common cause of persistent disability claims [4,5].

Studies in Quebec [6] and the Netherlands [7] suggest that early intervention using *participatory ergonomics *and return-to-work (RTW) coordination whose primary focus is the workplace, may hold promise in reducing disability and improving RTW following an episode of LBP. In these studies an ergonomist and/or occupational physician coordinate RTW by identifying injured worker and workplace barriers to RTW. This is followed by a meeting at the workplace with the injured worker and workplace parties with the goal of identifying solutions to the identified RTW barriers and devising a RTW plan. This approach demonstrated a two fold improvement in RTW compared to clinical interventions.

Recent systematic reviews [8,9] have also suggested that participatory ergonomics and RTW coordination are important elements in RTW. However workplace interventions are not well defined in this literature [10], including the two studies from Quebec and the Netherlands, which makes the interventions difficult to replicate. Moreover, the design and implementation of a workplace RTW intervention or program is dependent on jurisdiction. Although the workplace interventions in Quebec and The Netherlands appear similar they were tailored and implemented in the context of their respective settings. In The Netherlands, for example, there is no distinction between a work and non work related injury and sickness. They are both covered under the national disability insurance system with unique obligations required from the employer, injured person, health care provider and other stakeholders. In Canada, each province, including Quebec has its own workers' compensation system which differs in policies, procedures and practices.

Even within one jurisdiction, workplace RTW interventions are complex to design and implement. This is because of the many different workplace settings and stakeholders that exist, each with their own unique circumstances and the potential to impact RTW. Personnel used to implement and coordinate RTW interventions also differ depending on setting. Some settings use occupational physicians and ergonomists where others use nurses, health & safety consultants, RTW coordinators and/or RTW specialists. All these factors make designing workplace RTW interventions challenging.

Intervention mapping is a methodology used for designing and implementing complex interventions or programs. It has been used for over 20 years for systematically designing multifaceted programs involving numerous interventions directed at various individuals and environments [11]. Although traditionally used to develop community health promotion and disease prevention programs such AIDS prevention [12] and smoking cessation programs [13], intervention mapping is well suited for designing a workplace RTW program. This is because workplace RTW programs are also complex, necessitating a tailored and multifaceted approach directed at various stakeholders and settings [14].

The purpose of this study was to design a detailed workplace RTW program tailored to the Ontario setting using intervention mapping. The aim of RTW program is to reduce the duration of time off work and improve the sustainability of RTW following work-related LBP disability.

## Methods

**Figure 1:**
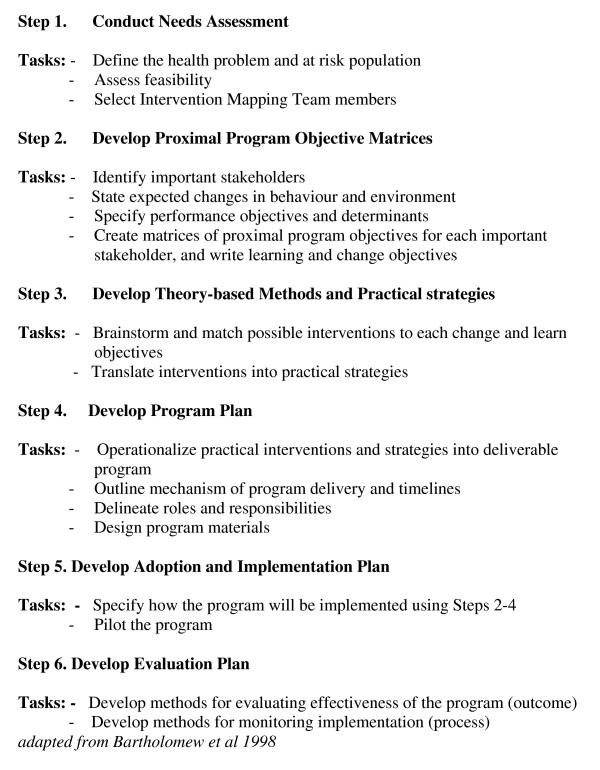
**Intervention Mapping Framework**.

The study design is a qualitative synthesis using the intervention mapping methodology as described by Bartholomew et al [11]. There are six steps in Intervention mapping. Step 1 consists of a needs assessment; steps 2, 3 and 4 involve the initial development of the intervention; step 5 consists of planning for implementation; and step 6 involves evaluation and refinement of the intervention. Figure 1 depicts the intervention mapping framework.

Within each step of intervention mapping, specific tasks are performed and questions answered which guide the decision making process. These tasks are accomplished systematically using *core processes *[11]. Core processes involve brain-storming among a selected group of individuals (known as the intervention mapping team made up of researchers, content experts and stakeholders), who come up with provisional solutions to the specific tasks and questions. This is achieved by consensus following a review of the literature for the best available evidence and theories around RTW and the management of occupational LBP, and combining this with the practical experiences of stakeholders. Example of questions included: *what does a work supervisor have to do to facilitate RTW in a worker with LBP? What are the determinants that will impact the supervisor's ability to facilitate successful RTW? What needs to change at the level of the supervisor in order to facilitate successful RTW?*

Best evidence was identified and comprised of systematic reviews [9,15-24], clinical practice guidelines [25-27] and key articles on prognosis and management of LBP and RTW [28-36]. We also identified and used relevant randomized controlled trials (RCT) aimed at improving RTW in occupational LBP [18,33-47]. Theories identified were those that filled evidence gaps and provided theoretical models for improving RTW, managing LBP and changing behaviours. These included participatory ergonomics [48-50], biopsychosocial [51] and cognitive behavioural [52-54] theories respectively.

Although presented as steps, the intervention mapping process is iterative, rather than linear, with the ability to move between steps and tasks as additional insight is gained during the process. Below is an outline of each of the intervention mapping steps.

### Step 1. Conduct a Needs Assessment

The objective of the needs assessment was to establish the rationale to improve RTW for occupational LBP in Ontario. It was also to assess the feasibility of performing the intervention mapping process at the Toronto Western Hospital. This is a University of Toronto teaching hospital and provides assessment and treatment, including workplace interventions for injured workers. 	The needs assessment was conducted by the principal investigator (CA) and co-investigators (DC, EB, PC) by examining trends in the duration of lost time claims in Ontario and meeting with Directors of the RTW Branch of the WSIB. The feasibility was assessed following discussions with the Directors of Research and Clinical Care at the hospital. The needs assessment also established the population of interest, which was the adult worker who suffers an occupational LBP episode and remains off work (acute and sub-acute LBP).

Following the needs assessment, an intervention mapping team was assembled consisting of three researchers, three RTW coordinators (RTWc), a behavioural therapist, an occupational physician, a WSIB physician and two physiotherapists. The team members were selected based on their experience in work-related disability and RTW and the ability to commit to the time obligations of the project. The intervention mapping team met biweekly for approximately nine months. Using a group discussion format the team worked collaboratively through the remaining intervention mapping steps and core processes.

### Step 2. Develop program objectives

The first task for the intervention mapping team was to use the core processes outlined above and list all important stakeholders that can impact RTW.	This was followed by listing performance objectives and expected outcomes for each identified stakeholder. Performance objectives are necessary activities that each stakeholder should perform to aid RTW. For example, for each stakeholder the intervention mapping team would answer the questions; *what do they (stakeholders) have to do to facilitate RTW and what are the expected outcomes for these actions? *Each performance objective was then matched with modifiable determinants that act as barriers or facilitators for achieving the objective. Determinants were grouped into three broad categories: cognitive-behavioural (attitudes, beliefs and emotions), knowledge and skills/self-efficacy. For the injured worker for example, one performance objective is the worker attempts to RTW on modified duties [8,27]. Fear of re-injury was listed as a potential cognitive-behavioral barrier [25], the lack of understanding between hurt and harm pain [2] was listed as a knowledge barrier and passive coping [55] was considered a skill/self-efficacy barrier to achieving this performance objective. Using the list generated for performance objectives and matching list of determinants, a matrix (performance objectives vs. determinants) was constructed for each stakeholder. In the body of the matrix, who and what needs to change and/or be learned (known as learn/change objectives) to achieve the objectives was outlined. For the injured worker for example, reducing fear of re-injury, understanding hurt vs. harm pain and avoiding passive coping were listed as change/learn activities in the body of the matrix, intersecting their respective performance objective and corresponding determinant. A matrix for each stakeholder group (injured worker, workplace, health care provider, WSIB and social network) was constructed.

The goal of step 2 was to identify for each important stakeholder all potential barriers and facilitators to RTW and their corresponding change and/or learned objectives. Examples of the injured worker and workplace matrices are summarized in Tables 1 and Additional file 1.

**Table 1:** Step 2. Matrix for injured worker: What does the injured worker need to do to return to work?

**Performance Objectives(worker)**	**Attitudes/Beliefs/Emotions**	**Knowledge**	**Skills/self-efficacy**	**Expected outcomes**
Keeps active despite pain and attempts RTW on modified work	Not fearful of re-injury	Understands difference between hurt and harm pain	Avoids passive coping	Demonstrates activity despite pain (avoidance of pain behaviours) and returns to modified work duties

Minimizes sitting or lying down	Positive attitude that avoiding sitting and lying will speed recovery			Avoids excessive sitting/lying down

Uses medication to control pain	Belief that medication can help with pain while returning to work	Learns coping/pacing strategies to control symptoms	Takes medication appropriately	Takes medication/performs exercise to reduce pain

Focus on function rather pain	Belief that the pain will subside. Has positive expectation	Understands the natural history of condition	Use proper body mechanics	

Co-operates with RTW co-coordinator/employer/supervisor	Trust in RTW coordinator			Avoids delay in RTW

Communicates with workplace re: job concerns	Belief that has a say in RTW process. Belief that employer will listen and understand concerns and is supportive	Learns how to make workplace safe	Develops sense of control at work. Can adapt/change situations at work.	Does not wait until 100% to RTW. Accepts reasonable RTW plan

Communicates with Health Care Providers	Belief that he/she is ready to RTW			Avoids delays in RTW with minimal and safe RTW restrictions

RTW = return to work

### Step 3. Develop theoretical methods and practical strategies

In step 3, the intervention mapping team generated a list of possible interventions that were matched to each change and/or learned objective listed in step 2. Evidence from the literature (clinical guidelines [25-27], systematic reviews [9,15-24], and key primary studies [18,33,34,37-47]) on interventions for acute and sub-acute LBP were compared and added to the list. Using theory, evidence, experience and consensus, a list was constructed of the most practical ways to implement these interventions. An attempt was made by the intervention mapping team to anchor the practical strategies to evidence based interventions. For the injured worker for example, to reduce fear of re-injury, a cognitive behavioural intervention [52] was listed as a potential intervention, and graded activity with positive re-enforcement [42] was recommended as a practical strategy. Providing the workplace with information and education about the importance of work accommodation and early RTW [8] was listed as an intervention. This intervention was translated into having a meeting at the workplace with the injured worker, supervisor and RTW coordinator to identify solutions for accommodation [8] as a practical strategy. Additional file 2 provides a table with further examples of practical strategies for interventions matched to change and/or learned objective for the workplace.

### Step 4. Design a workplace intervention program

The practical interventions and strategies compiled in step 3 were then operationalized into a deliverable RTW program with discrete components, mechanisms of delivery and timelines. This was achieved by the intervention mapping team using the core processes. The core processes were informed by interviews with RTW coordinators employed by the hospital. Hospital RTW coordinators are specifically trained and work primarily with injured workers who suffer from chronic pain. Their focus is to coordinate RTW for injured workers who appear capable and have a job to return to. The RTW coordinators provided valuable insight on practical logistics and sequencing of RTW intervention strategies. We also had the opportunity to observe experienced RTW coordinators in the field and documented their step by step activities.

We also conducted nine focus groups with the following stakeholders: injured workers, small employers (less than 30 employees), large employers, WSIB adjudicators and case managers, union representatives, RTW coordinators, physicians, chiropractors and physiotherapists and health and safety consultants. At each focus group session a preliminary draft the RTW program was presented. A focus group moderator detailed each step and asked for feedback on appropriateness and feasibility of the RTW program and how to best modify the program to maximize success of implementation. Each focus group session was audio recorded and transcribed. Summaries of the transcriptions were performed independently by two researchers (CA, SS) then synthesized via consensus. The synthesized feedback was then presented to the intervention mapping team. Using the core processes the intervention mapping team further refined the RTW program based on the synthesized feedback from the focus groups. The final step by step RTW program is outlined in Addition file 3.

Intervention mapping **Step 5 – Planning for program implementation **– involves repeating step 2 using performance objectives specific for program implementation (rather than design) and **Step 6 – Evaluation of the program **– involves testing the designed program in a real world setting. These will be performed in future studies.

Ethics approval was obtained from the University Health Network Research Ethics Review Board.

## Results

The intervention mapping team identified the following important stakeholders in RTW: 1) workplace stakeholders (employer, supervisor, union representative and health and safety consultant) 2) the injured worker 3) health care providers 4) WSIB adjudicator and nurse case manager and 5) family and social network including co-workers. RTW stakeholders are individuals or groups of individuals who have the capacity to either hamper and/or facilitate RTW.

The end result of the intervention mapping process was a comprehensive 5 Step RTW program. (See Additional file 3) A key finding of this process was that someone must take control and coordinate RTW. The RTW program (intervention) begins when barriers to RTW are identified by the insurer, the treating clinician or the workplace. The person or persons whose role is to coordinate RTW is then requested to intervene with the authorization and consent of the injured worker, third party payer and workplace. In our setting a RTW coordinator employed by the hospital and contracted by the WSIB, takes on this role and directs the RTW program. However, this role can be taken on by someone at the workplace, by the insurer or external consultant. For purpose of this study we refer to this person as the RTW coordinator.

The 5 Step RTW program is summarized as follows:

### RTW Program

#### Step I. Identifying barriers to RTW

The first task of the RTW program is to identify potential barriers to RTW from the perspective of all stakeholders who can impact RTW. This begins with the RTW coordinator interviewing the injured worker then the other stakeholders.

Prior to the interview, the injured worker completes self report questionnaires that assess the injured worker's pain, disability and potential psychosocial barriers to RTW such as anxiety, depression, aberrant coping and catastrophizing [56-58].

##### i) Interview with the injured worker

Barriers to RTW are assessed from the point of view of the injured worker. The injured worker is asked to list and rank RTW barriers from his or her perspective from most to least important. Regardless of how trivial a barrier is if it is deemed important to the injured worker then it is documented. During the interview, the RTW coordinator attempts to develop a good rapport, making the injured worker feel at ease. From the consultation and results of the self-report questionnaires, potential psychosocial barriers to RTW such as fear of re-injury, passive coping and catastrophizing are assessed. The injured worker is also asked about her/his relationships with the workplace, particularly her/his supervisor and co-workers. The supervisor and key decision makers at the workplace are identified by the injured worker. Finally, the injured worker is encouraged to contact the union representative and involve her or him in the RTW process.

##### ii) Interview with the third party payer

The third party payer is contacted. In the case of Ontario this would be the WSIB. Barriers to RTW from the perspective of the WSIB Case Manager are identified and ranked. If a lack of, or miscommunication between the WSIB and the injured worker is identified as a barrier, the RTW coordinator may suggest a teleconference between the injured worker, the WSIB Case Manager and the RTW coordinator. A copy of the Physical Demands Analysis (PDA) for the injured worker's job, if available, is requested from the WSIB Case manager. The PDA outlines the injured worker's pre-accident work duties.

##### iii) Interview with the Health Care Provider

The treating health care provider(s) are contacted and again asked to identify and rank barriers to RTW from their perspective. A copy of a Functional Abilities Form is obtained from the principal health care provider (usually the family doctor). In Ontario, the Functional Abilities Form is completed by the attending health care provider and outlines any restrictions for the injured worker when returning to work. This form is used to assist the employer in providing the injured worker with suitable work based on functional abilities.

##### iv) Interview with workplace

The supervisor and key decision makers (occupational health and safety person, human resources representative or disability manager) are contacted by the RTW coordinator and asked to identify and rank barriers to RTW. The injured worker's pre-injury job performance is discussed as well as the workplace's willingness to engage in RTW coordination including work modifications. The Physical Demands Analysis and the Functional Abilities Form are reviewed for any other potential barriers to RTW. A date is then set for a workplace meeting with injured worker, union representative, RTW coordinator and workplace parties.

#### Step II. Identifying solutions to RTW

##### i) Meeting at the Workplace

Prior to the meeting, the RTW coordinator outlines the ground rules for the meeting and emphasizes the importance of privacy and confidentiality and that only issues related to facilitating RTW will be discussed.

The RTW coordinator facilitates and engages communication between each stakeholder member, addressing the prioritized barriers to RTW. Each party is asked to come up with solutions to these barriers and then rank them based on importance and feasibility. The RTW coordinator facilitates consensus around practical solutions for RTW, who will be responsible for implementing these solutions, and a time line for implementation. Shared responsibility for the implementation of the agreed upon solutions is emphasized. A final RTW plan and tentative RTW date is mediated by the RTW coordinator.

##### ii) Tour worksite

Following the meeting, the injured worker and workplace decision makers tour the worksite and discuss agreed upon modifications and accommodations. The work demands are assessed and any perceived safety issues from the worker's perspective addressed. It is the responsibility of the workplace and the injured worker that work is conducted in a safe manner. The agreed RTW plan is reaffirmed. It is important that the injured worker feels empowered by the RTW process and that her/his reasonable concerns are addressed.

#### Step III. Preparation and implementation of RTW plan

The RTW coordinator writes a report outlining the agreed RTW plan. A copy of the report is provided to the injured worker and workplace representatives. A copy of the report is also sent to the third party payer (WSIB) and the health care providers for approval.

At each step of the RTW program the RTW coordinator maintains ongoing contact with the injured worker and provides reassurance [25,26]. If underlying psychosocial barriers were identified, such as fear of re-injury, activity avoidance or pain catastrophizing, then cognitive behavioural interventions [52] may be provided by the RTW coordinator (if qualified) or by a qualified team member. In the hospital setting, the RTW coordinator is part of a multidisciplinary team (which includes cognitive behavioural therapists and psychologists) working with injured workers. However, these interventions may be provided by the insurer or workplace. Cognitive behavioural interventions include graded activity, exercise, positive reinforcement, distraction and imagery.

#### Step IV. Implementing RTW solutions

##### i) Injured worker returns to work

The RTW coordinator contacts the workplace and the injured worker to determine whether the injured worker has returned to work at the agreed upon time, and performs the agreed upon duties. If necessary the RTW coordinator provides ongoing reassurance, positive reinforcement and education on self-management skills [59].

##### ii) Follow-up contact and/or discharge

Contact with the injured worker and workplace is maintained by the RTW coordinator following RTW and adjustments to the RTW plan are made to accommodate new information or overcome new barriers. The timing and intensity of the follow-up is tailored to the needs of the injured worker and workplace. The aim of the RTW coordinator is also to educate the injured worker and workplace on how to they can work together to resolve outstanding issues and future conflict. When issues are deemed too difficult or the parties have failed to resolve them then the RTW coordinator will intervene at the request of the workplace and/or injured worker.

The injured worker is discharged from the RTW program when, from the perspective of the injured worker and the workplace there are no significant barriers preventing sustainable RTW.

#### Step V. Evaluation of RTW plan

##### i) Document solutions implemented

During the initial follow-up contact, an assessment is made from the perspective of the injured worker and the workplace party (supervisor), on whether each agreed solution in the RTW plan was implemented fully, partially or not at all. Satisfaction with the RTW plan among stakeholders is also assessed using a 5-point Likert scale ranging from very satisfied to very dissatisfied. This assessment is administered face to face at the workplace or by e-mail, fax or phone.

##### ii) Write progress report

A final report is sent to the third party payer (WSIB), injured worker, the workplace and health care providers.

## Discussion

In this study, we describe the step-by-step development of a comprehensive RTW program for occupational LBP. To our knowledge this is the first study to outline the design of a comprehensive RTW program for occupational LBP using an intervention mapping approach. Designing RTW programs are inherently challenging because of the complex and multi-faceted nature of RTW.

Intervention mapping provides a very useful framework to systematically guide us through this complexity. Of particular importance is the input from stakeholders who provide practical strategies to improve RTW. Another important feature of the intervention mapping process is the ability to tailor the interventions within the RTW program to the needs of various stakeholders and environments. A major drawback to the intervention mapping approach is that it is very time consuming and resource intensive.

The impetus for this study was two previous studies, one from Quebec [6] and the other from the Netherlands [7]. These studies developed and tested RTW interventions based on participatory ergonomics and RTW coordination. These studies demonstrated a two fold improvement in time off work due to occupational LBP compared to usual care. Although the details of the RTW interventions were not well described in these and other studies [10], evidence-based RTW programs need to be adapted and contextualized to a specific jurisdiction and setting. In Ontario, differences in workers' compensation regulations, labour laws and health care systems would make replicating RTW programs implemented in other jurisdictions problematic. The intervention mapping approach used in this study enabled the design of a RTW program tailored to the Ontario setting. This methodology can be used to develop and tailor RTW programs in other jurisdictions.

Although the focus of our RTW program is the workplace, similar to the Quebec and The Netherland studies, our RTW program differs in that it identifies and addresses RTW barriers beyond the workplace. These include communication barriers with the third party payer or health care practitioner(s) and potential underlying psychosocial barriers involving the injured worker and his/her social environment. To address these other barriers, the individual(s) coordinating and implementing the RTW program, such as a RTW coordinator will require diverse skills, including the ability to engage in cognitive behavioural treatment (or coaching) or have access to other qualified professionals who can perform these activities. In addition to developing a comprehensive RTW program, another important contribution of this study is that we have outlined the roles and responsibilities involved in RTW coordination. RTW coordination can be performed by the insurer, health care provider (occupational nurse or physician) or disability manager provided they have the skills and knowledge to perform the duties outlined in the RTW program. RTW coordination does not necessarily mean a comprehensive intervention is needed. Sometimes all that may be required is a simple phone call. But someone needs to identify that a lack of communication exists and that a phone call is needed. 	

Most injured workers return to work without the need of assistance. Among those who remain off work for reasons beyond the physical impairment of the injury, barriers are usually organizational and psychosocial in nature. When there is a discrepancy between the injury and ability to work then RTW coordination can be useful.

In our setting, RTW coordinators have a health care background (occupational and/or physical therapist or kinesiologist) with basic skills in ergonomic assessment and modifications. They also have informal training in cognitive behavioural coaching provided by cognitive behavioural therapists and psychologists who are members of the team. It is expected that in a sub acute LBP population, extensive knowledge in these areas is not often necessary. Physical therapists have been trained to perform basic cognitive behavioural therapy and can obtain similar outcomes compared to psychologists [60]. In term of ergonomic expertise, the Netherland study demonstrated that less than half of the recommended ergonomic solutions where actually implemented suggesting that the process of engagement and shared decision making was more important than the actual ergonomic changes [7,61].

There are several important strengths of this study. We used a comprehensive and systematic approach (intervention mapping) in the design of the RTW program. Although this approach is novel in the occupational setting intervention mapping has been used extensively in the design of complex community health programs for over 20 years [11]. In addition to using the best available evidence, the approach is participatory. We consulted and received feedback from all important stakeholders throughout the development of the RTW program, who contributed practical insight on what works and what doesn't in RTW. This process also ensured that our program was relevant not to one but to all important stakeholders. We also had the opportunity to witness RTW coordinators in the field which added further insight on how to design a practical program. Finally, our RTW program, and the roles and responsibilities of RTW coordination, are explicitly detailed yet can be tailored to various settings, leading to wider applicability of the RTW program.

Potential weaknesses of the study include the design of the program within a hospital academic setting and using hospital based RTW coordinators who typically manage a more chronic population. This would potentially narrow the generalizability and applicability of our RTW program. Although designed in a hospital setting the draft RTW program was refined and contextualized with consultation and feedback from nine different stakeholder groups outside the hospital setting. In addition, the evidence and theories that formed the basis of the interventions within the RTW program focused on sub-acute LBP and not chronic pain. The RTW program in its design can be tailored to the various settings and injured worker populations. The underlying principles of the RTW program likely apply regardless of duration of symptoms, location of injury or setting.

During the design of the RTW program, the WSIB in Ontario was under going a change in their service delivery model with associated restructuring of their policies and procedures. This may also limit its applicability. However, the principals of RTW program should still apply although the delivery of the RTW program may have to be modified. Moreover, employers and other third party payers can use the RTW program to develop their own RTW program, adapting it to their setting and aligning it with the new WSIB service delivery model.

The subjective nature of interpretation of the evidence, theories and experiences of stakeholders may result in a different RTW program depending on the make-up and biases of the intervention mapping team members. However, the final RTW program and essential elements are consistent with current high quality systematic reviews and other published workplace RTW programs. Finally the effectiveness of the RTW program has not yet been evaluated.

Ideally preventing work-related LBP is preferable to attempting to prevent LBP disability after an injury but this is not always feasible or effective [17,62]. However, primary and secondary prevention have been shown to be linked. Interventions directed at secondary prevention can impact primary prevention outcomes and visa a versa [63,64]. Comprehensive approaches that incorporate both primary and secondary prevention strategies should be considered.

## Conclusion

We have a developed a 5 step RTW program to improve RTW in Ontario for injured workers with sub-acute LBP using an intervention mapping approach. The next step will be to evaluate the RTW program. We plan to pilot test the RTW program in Ontario workplaces. This will be followed by an evaluation of the effectiveness and cost-effectiveness of the RTW Program. We also plan to compare the RTW program to those implemented in the Quebec and The Netherland studies. Finally, we plan to further develop and test the methodology used in this study to adapt the RTW program to other jurisdictions in Canada and around the world.

## Competing interests

The authors declare that they have no competing interests.

## Authors' contributions

CA conceived and coordinated the study and drafted the manuscript. DC participated in the design and coordination of the study and helped draft the manuscript. IS participated in implementation of the study and assisted in drafting the manuscript. SS facilitated the focus group sessions and lead the synthesis of the qualitative data. EB participated in the design, writing the ethics application and drafting the manuscript. SE mapped out procedures performed by RTW coordinators and participated in intervention mapping team meetings. HH participated in the intervention mapping team meetings and provided technical guidance. BB assisted in interpretation of the data. PC assisted in the design of the study and helped write the manuscript. All authors read and approved the final manuscript.

## Pre-publication history

The pre-publication history for this paper can be accessed here:



## Supplementary Material

Additional file 1 **Step 2. Matrix for workplace: What the workplace needs to do to improve RTW?**. the table describes a matrix where performance objectives of the injured worker are matched to corresponding determinants. The body of the matrix outlines what need to be learned or changed in order to achieve the performance objectives. Click here for file

Additional file 2 **Step 3. Intervention methods and strategies for the Workplace**. the table describes the translation of learned and change objectives for the workplace into interventions and practical strategies Click here for file

Additional file 3 **Step 4. Operationalize RTW Workplace Interventions into RTW Program**. The table outlines a comprehensive step by step RTW Program Click here for file
