# Supplementary material for: Designing a workplace return-to-work program for occupational low back pain: an intervention mapping approach
Source: BMC Musculoskelet Disord. 2009 Jun 9;10:65. doi: 10.1186/1471-2474-10-65 (PMC2700788; doi:10.1186/1471-2474-10-65)
Supplement: Additional file 1 — Step 2. Matrix for workplace: What the workplace needs to do to improve RTW?. the table describes a matrix where performance objectives of the injured worker are matched to corresponding determinants. The body of the matrix outlines what need to be learned or changed in order to achieve the performance objectives. [file 1471-2474-10-65-S1.doc]

| **Performance**  **Objectives (workplace)** | **Attitudes/**  **Beliefs/ Emotions** | **Knowledge** | **Skills/self-efficacy** | **Expected outcomes** |
| --- | --- | --- | --- | --- |
| Willingness to meet with  worker at workplace | Positive attitude about getting worker back to work |  |  | Supervisor schedules time to meet with worker at workplace |
| Willingness to listen to workers concerns  (open communication) | Shows empathy and concern, not confrontational |  | Learning to listen to worker concerns |  |
| Willingness to act on reasonable suggestions from worker |  | understands that addressing worker concern improves RTW |  | Helps prioritizes accommodations |
| Accommodates workplace to facilitate RTW for worker/ adapt to workers needs  (Ergonomic evaluation/design) | Positive attitude about making changes to assist worker. Willingness for shared responsibility in making RTW successful |  | Demonstrates ability to adapt worksite to suit worker | Helps in the development of time table for change |
| Provides flexibility and some control for work breaks/pacing |  | Explain that increase decision latitude make for healthy workplace |  | Worker is able to pace and take breaks (increase control) |
| Makes suitable work available |  | Explains impact of worker fear of re-injury | Demonstrates ability to adapt worksite to facilitate worker’s RTW |  |
| Supervisor show concern and provide support for worker | Positive attitude about worker’s RTW and accommodations | Explains to co-workers the need to support injured worker |  | Co-workers/supervisor  Provide support for worker |
| Shared responsibility for worker and supervisor | Positive attitude about input from worker on RTW |  |  | Joint effort by supervisor and worker on facilitating RTW |
| Supervisor ensures that solutions for RTW do not interfere with co-workers jobs |  |  |  | Co-workers not overly inconvenienced due to injured worker’s RTW |
| Follow-up needed to check RTW schedule | Positive attitude about the need for time contingency for successful RTW |  |  | Follow-up conducted on changes needed/ requested/ negotiated and RTW plan |

RTW = Return-to-work
