# Supplementary material for: Designing a workplace return-to-work program for occupational low back pain: an intervention mapping approach
Source: BMC Musculoskelet Disord. 2009 Jun 9;10:65. doi: 10.1186/1471-2474-10-65 (PMC2700788; doi:10.1186/1471-2474-10-65)
Supplement: Additional file 2 — Step 3. Intervention methods and strategies for the Workplace. the table describes the translation of learned and change objectives for the workplace into interventions and practical strategies [file 1471-2474-10-65-S2.doc]

| Determinants and  change objective | Intervention | Strategy |
| --- | --- | --- |
| Positive attitude about getting worker back to work | Provide information/education about early RTW and LBP addressing negative/false attitudes and beliefs and knowledge. Stress communication and cooperation. Return to regular duties as soon as possible | Research the workplace. Early telephone contact with workplace to set up meeting. Interview employer. Provide information on RTWc role and explain process |
| Knowledge about early RTW for LBP | Provide information/education about early RTW and LBP addressing negative/false attitudes and beliefs and knowledge Stress communication and cooperation. Return to regular duties as soon as possible | Communication at the workplace with RTWc. Provide educational material on RTW |
| Ability to listen to RTWc and injured worker about workplace concerns | Provide information/education about early RTW and LBP addressing negative/false attitudes and beliefs and knowledge Stress communication and cooperation. | RTWc discusses with supervisor importance of shared responsibility and communication with worker |
| Positive attitude about making changes to assist worker’s RTW. | Provide information/education about early RTW and LBP addressing negative/false attitudes and beliefs and knowledge Stress communication and cooperation. | RTWc observes the workplace and documents any all possible barriers. Compares worker perceived abilities and workplace physical demands. Discusses problems with RTW of injured worker |
| Ability to adapt worksite to suit worker  Positive attitude about overcoming problems/risks. Modify high physical work demand tasks (temporally) | Provide information/education about early RTW and LBP addressing negative/false attitudes and beliefs and knowledge. Stress communication and cooperation. | Meeting with RTWc, injured worker and supervisor at workplace. Discuss concerns and identify all possible solutions for RTW. Advice on graded activity and goal setting. Assist In problem solving. |
| Positive attitude about increased decision latitude of worker (pacing) | Provide information/education about early RTW and LBP addressing negative/false attitudes and beliefs and knowledge. Increase worker decision latitude Stress communication and cooperation. | Communicates with supervisor about ability to pace and increased decision latitude |
| Ability to explain to co workers to obtain their support for RTW of injured worker | Provide information/education about early RTW and LBP addressing negative/false attitudes and beliefs and knowledge. Stress communication and cooperation among all parties | Discuses with supervisor to have meeting with co-workers to discuss RTW and to provide support |
| Positive attitude/support of Union for early RTW | Provide information/education about early RTW and LBP addressing negative/false attitudes and beliefs and knowledge. Stress communication and cooperation among all parties | RTWc meets with Union and discusses RTW process and role of RTWc |
| Positive attitude about shared responsibility for RTW | Provide information/education about early RTW and LBP addressing negative/false attitudes and beliefs and knowledge. Stress communication and cooperation among all parties | Discusses with union/supervisor and worker importance of shared responsibility for RTW |
| Ability to ensure that RTW solutions do not interfere with co-workers | Provide information/education about early RTW and LBP addressing negative/false attitudes and beliefs and knowledge. Stress communication and cooperation among all parties | RTWc with worker, union and supervisor discuss solutions that minimize interference with co-workers |
| Ability to provide transitional/ modification among small/family businesses | Provide information/education about early RTW and LBP addressing negative/false attitudes and beliefs and knowledge. Stress communication and cooperation among all parties | RTWc discusses ways to integrate worker back to work while minimizing financial strain. May also involve contact with WSIB. |
| Positive attitude and ability to provide time limited transitional duties | Provide information/education about early RTW and LBP addressing negative/false attitudes and beliefs and knowledge. Stress communication and cooperation among all parties and return to regular duties | RTWc discusses with union/worker/supervisor the importance of minimizing modified/transitional duties and to return to regular duties in specified time period |
| Positive attitude about setting a RTW date and making changes to workplace | Provide information/education about early RTW and LBP addressing negative/false attitudes and beliefs and knowledge. Stress communication and cooperation among all parties and return to regular duties | RTWc, worker and supervisor decide on a RTW date and confirm timetable for necessary changes at workplace |

RTW = return to work, RTWc = return to work coordinator, LBP = low back pain
