# Supplementary material for: Designing a workplace return-to-work program for occupational low back pain: an intervention mapping approach
Source: BMC Musculoskelet Disord. 2009 Jun 9;10:65. doi: 10.1186/1471-2474-10-65 (PMC2700788; doi:10.1186/1471-2474-10-65)
Supplement: Additional file 3 — Step 4. Operationalize RTW Workplace Interventions into RTW Program. The table outlines a comprehensive step by step RTW Program [file 1471-2474-10-65-S3.doc]

| STEPS | ACTIVITIES OF RTW COORDINATION |
| --- | --- |
| 1. Identifying barriers to RTW | 1. Interview the injured worker:  - obtain history of injury, occupational information, employment history, past claims, health information and employer information. Review injured worker self report questionnaires - build therapeutic rapport, assess therapeutic needs (physical and psychosocial), and assess relationship with employer and perception of job demands - list and rank RTW barriers from injured worker’s perspective provide education and reassurance - obtain names of contact individuals at workplace, supervisor and decision maker - obtain consent to contact employer/Union  1. Contact with WSIB case manager (by phone):    - discuss history of claim, and establish working relationship and collaboration.  - obtain authorization for worksite assessment - list and rank RTW barriers from WSIB case manager’s perspective - obtain PDA (physical demands analysis) if available  1. Contact the employer (by phone):    - contact appropriate decision maker at worksite    - discuss pre-injury job performance    - list and rank barriers to RTW from employer’s perspective Confirm PDA and need of FAF (functional abilities form from health care provider).  - obtain contact information of direct supervisor - contact supervisor and union rep. List and rank barriers to RTW from their perspective - request meeting at workplace with injured worker, decision maker, supervisor and union rep. Confirm meeting date and time.   e) Contact health care provider(s) and family doctor (by phone)   - list and rank barriers to RTW from the health care provider’s perspective - request Functional Abilities Form (FAF) if necessary |
| 2. Identifying solutions for RTW | 1. Meeting at workplace:  - mediate the meeting with the injured worker, workplace decision maker, supervisor and union rep   - ensure privacy and confidentiality (discuss RTW related issues only)   - group similar barriers obtained in Step 1. Obtain group consensus on high to low priority barriers.   - ask each member to list all possible solutions for each prioritized barrier and obtain consensus on high to low priority solutions   - discuss the feasibility and practicality of implementing solutions   - get group consensus on high priority and feasible solutions   - discuss who will be responsible and accountable for implementing RTW solutions (shared responsibility)   - facilitates the negotiation of a RTW plan and tentative RTW date   - confirm date for follow-up meeting   - document agreed solutions and negotiated RTW plan  1. Tour worksite with injured worker/supervisor/decision maker:  - assess safety - assess work demands - review/assess agreed modifications (solutions) and their implementation - identify other possible solutions - recommit to RTW plan and follow-up meeting - ensure injured worker feels empowered by process - provide reassurance and positive reinforcement - document additional solutions and if required potential psychosocial strategies |
| 3. Preparation and implementation of RTW plan | 1. Write worksite report  - list goals and objectives of RTW plan - outline identified solutions - identify who will be responsible for implementing solutions - set time-table for implementation and RTW - include educational material - contact and discuss report with client and employer - obtain commitment from client and employer - provide psychosocial support (coaching) using cognitive behavioural approach where needed - document agreed implementation plan  1. Contact health care provider(s) (fax/phone):  - send copy of RTW report - educate on RTW - obtain support for RTW plan - document level of support from health care providers  1. Contact WSIB (fax/phone):  - send copy of RTW report - educate on RTW - obtain authorization to proceed with RTW plan - document authorization from WSIB |
| 4. Implementing RTW solutions | a) Injured worker returns to work:   - determines if the injured worker returns to work on specified time frame and duties - provide reassurance, positive reinforcement and educate on self-management skills  1. Follow-up contact and coaching:  - reinforce the RTW plan with all stakeholders. Set goals and objectives and time frames. - use cognitive behavioural approach to deal with potential psychosocial challenges - adjust of RTW plan to accommodate new information or overcome new barriers to RTW   d) Focus from attending clinical interventions to attending work:   - help to finalize or coordinate any ongoing care.   e) document solutions implemented: |
| 5. Evaluation of RTW plan | a) Compliance to the intervention:   - assess compliance to RTW program (compliance questionnaire)   b) Document solutions implemented:   - administer implementation questionnaire to injured worker and employer on solutions implemented - document whether solutions were implemented fully, partially or not at all   c) Feedback on RTW plan/intervention:   - administer satisfaction questionnaire to injured worker, employer, health care providers and WSIB (satisfaction with process and perceived effectiveness of RTW plan)   d) Write and send progress report to all stakeholders |

RTW = return to work, WSIB = Workplace Safety and Insurance Board
